# Notification that new names of prokaryotes, new combinations, and new taxonomic opinions have appeared in volume 74, part 2 of the IJSEM

**DOI:** 10.1099/ijsem.0.006301

**Published:** 2024-06-03

**Authors:** Aharon Oren, Markus Göker

**Affiliations:** 1 The Institute of Life Sciences, The Hebrew University of Jerusalem, The Edmond J. Safra Campus, 9190401 Jerusalem, Israel; 2 Leibniz Institute DSMZ – German Collection of Microorganisms and Cell Cultures, Inhoffenstrasse 7B, 38124 Braunschweig, Germany

This listing of names of prokaryotes published in a previous issue of the *International Journal of Systematic and Evolutionary Microbiology* (IJSEM) is provided as a service to microbiology to assist in the recognition of new names and new combinations. This procedure was proposed by the Judicial Commission [1]. The names given herein are listed according to the Rules of priority (i.e., time and date of publication of the articles on the journal’s platform and the order of valid publication of names in the original articles) [2].

**Table:** 

Order of article publication	Name/authors	Proposed as	Article no.	Page no.
1	*Paenibacillus glufosinatiresistens* Wu *et al*. 2024	sp. nov.	006259	7
2	*Chryseobacterium fluminis* Kim *et al*. 2024	sp. nov.	006261	6
3	*Flavobacterium odoriferum* Ran *et al*. 2024	sp. nov.	006258	8
3	*Flavobacterium fragile* Ran *et al*. 2024	sp. nov.	006258	9
3	*Flavobacterium luminosum* Ran *et al*. 2024	sp. nov.	006258	9
4	*Streptomyces herbicida* Luo *et al*. 2024	sp. nov.	006263	6
5	*Myceligenerans pegani* Li *et al*. 2024	sp. nov.	006179	7
6	*Tepidibacteraceae* Bello *et al*. 2024	fam. nov.	006247	19
6	*Peptoclostridiaceae* Bello *et al*. 2024	fam. nov.	006247	19
6	*Alkalithermobacter* Bello *et al*. 2024	gen. nov.	006247	19
6	*Alkalithermobacter paradoxus* (Li *et al*. 1993) Bello *et al*. 2024	comb. nov. [basonym: *Clostridium paradoxum* Li *et al*. 1993]	006247	20
6	*Alkalithermobacter thermoalcaliphilus* (Li *et al*. 1994) Bello *et al*. 2024*	comb. nov. [basonym: *Clostridium thermocalcaliphilum* Li *et al*. 1994]	006247	20
6	*Faecalimicrobium* Bello *et al*. 2024	gen. nov.	006247	20
6	*Faecalimicrobium dakarense* Bello *et al*. 2024	sp. nov.	006247	20
6	*Metaclostridioides* Bello *et al*. 2024	gen. nov.	006247	20
6	*Metaclostridioides mangenotii* (Prévot and Zimmès-Chaverou 1947) Bello *et al*. 2024	comb. nov. [basonym: ‘*Inflabilis mangenoti*’ Prévot and Zimmès-Chaverou 1947]†	006247	21
6	*Paraclostridium tenue* (Bergey *et al*. 1923) Bello *et al*. 2024	comb. nov. [basonym: ‘*Bacteroides tenuis*’ Bergey *et al*. 1923]§	006247	22
6	*Paraclostridium ghonii* (Prévot 1938) Bello *et al*. 2024	comb. nov. [basonym: *Clostridium ghonii* corrig. Prévot 1938 (Approved Lists 1980)]	006247	22
6	*Paraclostridium sordellii* (Hall and Scott 1927) Bello *et al*. 2024	comb. nov. [basonym: ‘*Bacillus sordelli’* (sic) Hall and Scott 1927]‡	006247	22
7	*Solibacillus palustris* Jo *et al*. 2024	sp. nov.	006065	5
8	*Anaeromyxobacter terrae* Tang *et al*. 2024	sp. nov.	006268	7
8	*Anaeromyxobacter oryzisoli* Tang *et al*. 2024	sp. nov.	006268	7
8	*Anaeromyxobacter soli* Tang *et al*. 2024	sp. nov.	006268	8
9	*Glycomyces niveus* Yang *et al*. 2024	sp. nov.	006265	6
10	*Paenibacillus baimaensis* Wang *et al*. 2024	sp. nov.	006260	6
11	*Speluncibacter* Lee *et al*. 2024	gen. nov.	006267	9
11	*Speluncibacter jeojiensis* Lee *et al*. 2024	sp. nov.	006267	11
11	*Speluncibacteraceae* Lee *et al*. 2024	fam. nov.	006267	11
12	*Erysipelothrix amsterdamensis* Zhong *et al*. 2024	sp. nov.	006264	7
13	*Amycolatopsis mongoliensis* Oyuntsetseg *et al*. 2024	sp. nov.	006266	7
14	*Chitiniphilus purpureus* Li *et al*. 2024	sp. nov.	006245	8
15	*Stenotrophomonas riyadhensis* Macori *et al*. 2024	sp. nov.	006250	3
16	*Polycladomyces zharkentensis* Mashzhan *et al*. 2024	sp. nov.	006269	8
17	*Aerococcus kribbianus* Bai *et al*. 2024	sp. nov.	006284	8
18	*Moraxella oculi* Wilkes *et al*. 2024	sp. nov.	006281	5
19	*Rodentibacter caecimuris* (Lagkouvardos *et al*. 2016) Li 2024	comb. nov. [basonym: *Pasteurella caecimuris* Lagkouvardos *et al*. 2016]	006279	3
20	*Roseibium algicola* Lee *et al*. 2024	sp. nov.	006283	7
20	*Roseibium porphyridii* Lee *et al*. 2024	sp. nov.	006283	7
21	*Lactobacillus juensis* Jiang and Gu 2024	sp. nov.	006285	5
21	*Lactobacillus rizhaonensis* Jiang and Gu 2024	sp. nov.	006285	8
22	*Flagellimonas abyssi* (Wang *et al*. 2022) Molinari Novoa *et al*. 2024	comb. nov. [basonym: *Muricauda abyssi* Wang *et al*. 2022]	006286	1
22	*Flagellimonas aequoris* (Guo *et al*. 2020) Molinari Novoa *et al*. 2024	comb. nov. [basonym: *Muricauda aequoris* Guo *et al*. 2020]	006286	2
22	*Flagellimonas allohymeniacidonis* Molinari Novoa *et al*. 2024	nom. nov. [basonym: *Muricauda hymeniacidonis* Park 2019]	006286	2
22	*Flagellimonas alvinocaridis* (Liu *et al*. 2020) Molinari Novoa *et al*. 2024	comb. nov. [basonym: *Muricauda alvinocaridis* Liu *et al*. 2020]	006286	2
22	*Flagellimonas amoyensis* (Li *et al*. 2019) Molinari Novoa *et al*. 2024	comb. nov. [basonym: *Muricauda amoyensis* Li *et al*. 2019]	006286	3
22	*Flagellimonas amphidinii* (Chen *et al*. 2021) Molinari Novoa *et al*. 2024	comb. nov. [basonym: *Muricauda amphidinii* Chen *et al*. 2021]	006286	3
22	*Flagellimonas antarctica* (Wu *et al*. 2013) Molinari Novoa *et al*. 2024	comb. nov. [basonym: *Muricauda antarctica* Wu *et al*. 2013]	006286	3
22	*Flagellimonas aurea* (Zhao *et al*. 2022) Molinari Novoa *et al*. 2024	comb. nov. [basonym: *Muricauda aurea* Zhao *et al*. 2022]	006286	3
22	*Flagellimonas beolgyonensis* (Lee *et al*. 2012) Molinari Novoa *et al*. 2024	comb. nov. [basonym: *Muricauda beolgyonensis* Lee *et al*. 2012]	006286	4
22	*Flagellimonas chongwuensis* (Chen *et al*. 2022) Molinari Novoa *et al*. 2024	comb. nov. [basonym: *Muricauda chongwuensis* Chen *et al*. 2022]	006286	4
22	*Flagellimonas flavescens* (Yoon *et al*. 2005) Molinari Novoa *et al*. 2024	comb. nov. [basonym: *Muricauda flavescens* Yoon *et al*. 2005]	006286	4
22	*Flagellimonas hadalis* (Zhang *et al*. 2020) Molinari Novoa *et al*. 2024	comb. nov. [basonym: *Muricauda hadalis* Zhang *et al*. 2020]	006286	4
22	*Flagellimonas indica* (Zhang *et al*. 2018) Molinari Novoa *et al*. 2024	comb. nov. [basonym: *Muricauda indica* Zhang *et al*. 2018]	006286	4
22	*Flagellimonas iocasae* (Liu *et al*. 2018) Molinari Novoa *et al*. 2024	comb. nov. [basonym: *Muricauda iocasae* Liu *et al*. 2018]	006286	5
22	*Flagellimonas lutaonensis* (Arun *et al*. 2009) Molinari Novoa *et al*. 2024	comb. nov. [basonym: *Muricauda lutaonensis* Arun *et al*. 2009 emend. Hahnke *et al*. 2016]	006286	5
22	*Flagellimonas lutimaris* (Yoon *et al*. 2008) Molinari Novoa *et al*. 2024	comb. nov. [basonym: *Muricauda lutimaris* Yoon *et al*. 2008]	006286	5
22	*Flagellimonas marina* (Su *et al*. 2017) Molinari Novoa *et al*. 2024	comb. nov. [basonym: *Muricauda marina* Su *et al*. 2017]	006286	5
22	*Flagellimonas marinaquae* Molinari Novoa *et al*. 2024	nom. nov. [basonym: *Muricauda aquimarina* Yoon *et al*. 2005]	006286	5
22	*Flagellimonas meishanensis* (Sun *et al*. 2023) Molinari Novoa *et al*. 2024	comb. nov. [basonym: *Muricauda meishanensis* Sun *et al*. 2023]	006286	6
22	*Flagellimonas meridianipacifica* Molinari Novoa *et al*. 2024	nom. nov. [basonym: *Muricauda pacifica* Zhang *et al*. 2015 emend. García-López *et al*. 2019]	006286	6
22	*Flagellimonas myxillae* (Moon *et al*. 2023) Molinari Novoa *et al*. 2024	comb. nov. [basonym: *Muricauda myxillae* Moon *et al*. 2023]	006286	6
22	*Flagellimonas nanhaiensis* (Dang *et al*. 2019) Molinari Novoa *et al*. 2024	comb. nov. [basonym: *Muricauda nanhaiensis* Dang *et al*. 2019]	006286	6
22	*Flagellimonas oceanensis* (Guo *et al*. 2020) Molinari Novoa *et al*. 2024	comb. nov. [basonym: *Muricauda oceanensis* Guo *et al*. 2020]	006286	6
22	*Flagellimonas oceani* (Dong *et al*. 2020) Molinari Novoa *et al*. 2024	comb. nov. [basonym: *Muricauda oceani* Dong *et al*. 2020]	006286	7
22	*Flagellimonas ochracea* (Kim *et al*. 2020) Molinari Novoa *et al*. 2024	comb. nov. [basonym: *Muricauda ochracea* Kim *et al*. 2020]	006286	7
22	*Flagellimonas okinawensis* (Cao *et al*. 2023) Molinari Novoa *et al*. 2024	comb. nov. [basonym: *Muricauda okinawensis* Cao *et al*. 2023]	006286	7
22	*Flagellimonas olearia* (Hwang *et al*. 2009) Molinari Novoa *et al*. 2024	comb. nov. [basonym: *Muricauda olearia* Hwang *et al*. 2009]	006286	7
22	*Flagellimonas onchidii* (Liang *et al*. 2021) Molinari Novoa *et al*. 2024	comb. nov. [basonym: *Muricauda onchidii* Liang *et al*. 2021]	006286	7
22	*Flagellimonas pelagia* Molinari Novoa *et al*. 2024	nom. nov. [basonym: *Muricauda maritima* Guo *et al*. 2020]	006286	8
22	*Flagellimonas profundi* (Zhao *et al*. 2022) Molinari Novoa *et al*. 2024	comb. nov. [basonym: *Muricauda profundi* Zhao *et al*. 2022]	006286	8
22	*Flagellimonas ruestringensis* (Bruns *et al*. 2001) Molinari Novoa *et al*. 2024	comb. nov. [basonym: *Muricauda ruestringensis* Bruns *et al*. 2001]	006286	8
22	*Flagellimonas sediminis* (Zhu *et al*. 2021) Molinari Novoa *et al*. 2024	comb. nov. [basonym: *Muricauda sediminis* Zhu *et al*. 2021]	006286	8
22	*Flagellimonas spongiicola* (Shin and Park 2023) Molinari Novoa *et al*. 2024	comb. nov. [basonym: *Muricauda spongiicola* Shin and Park 2023]	006286	9
22	*Flagellimonas taeanensis* (Kim *et al*. 2013) Molinari Novoa *et al*. 2024	comb. nov. [basonym: *Muricauda taeanensis* Kim *et al*. 2013 emend. Zhang *et al*. 2020]	006286	9
22	*Flagellimonas yonaguniensis* (Cao *et al*. 2023) Molinari Novoa *et al*. 2024	comb. nov. [basonym: *Muricauda yonaguniensis* Cao *et al*. 2023]	006286	9
22	*Flagellimonas zhangzhouensis* (Yang *et al*. 2013) Molinari Novoa *et al*. 2024	comb. nov. [basonym: *Muricauda zhangzhouensis* Yang *et al*. 2013]	006286	9

*Given as t*hermoalcaliphilum* in the protologue heading.

†The authors gave *Clostridium mangenotii* (Prévot and Zimmès-Chaverou 1947) McClung and McCoy 1957 (Approved Lists 1980) as basonym.

‡The authors gave *Clostridium sordellii* (Hall and Scott 1927) Prévot 1938 (Approved Lists 1980) as basonym.

§The authors gave *Eubacterium tenue* (Bergey *et al*. 1923) Holdeman and Moore 1970 (Approved Lists 1980) as basonym.

The following taxonomic opinions (i.e., the emendation of circumscriptions or the creation of synonyms) do not affect the status of a name under the International Code of Nomenclature of Prokaryotes. These taxonomic opinions can neither be considered as approved nor as disapproved by the International Committee on Systematics of Prokaryotes.

**Table:** 

Name/authors:	Article no.	Page no.
*Clostridioides* Lawson *et al*. 2016 emend. Bello *et al*. 2024	006247	21
*Flagellimonas* Bae *et al*. 2007 emend. Molinari Novoa *et al*. 2024	006286	1
*Paraclostridium* Sasi Jyothsna *et al*. 2016 emend. Bello *et al*. 2024	006247	21
*Peptostreptococcaceae* Ezaki 2010 emend. Bello *et al*. 2024	006247	21
*Rodentibacter heylii* Adhikary *et al*. 2017 pro synon. *Rodentibacter caecimuris* (Lagkouvardos *et al*. 2016) Li 2024	006279	3
*Romboutsia* Gerritsen *et al*. 2014 emend. Bello *et al*. 2024	006247	21
